# Sarcoidosis-like disease mimicking metastases during adjuvant ipilimumab therapy in advanced melanoma patient: CT scan and MRI help in managing difficult clinical decision

**DOI:** 10.1259/bjrcr.20190065

**Published:** 2020-09-29

**Authors:** Enrico Matteo Garanzini, Davide Scaramuzza, Gaia Spadarella, Lorenza Di Guardo, Alfonso Marchianò

**Affiliations:** 1 Postgraduation School of Radiology, Università degli Studi di Milano, Milan, Italy; 2 Department of Radiology, Fondazione IRCCS Istituto Nazionale dei Tumori, Milan, Italy; 3 Department of Medical Oncology, Fondazione IRCCS Istituto Nazionale dei Tumori, Milan, Italy

## Abstract

The onset of an autoimmune, sarcoidosis-like reaction during or after treatment with immunomodulatory drugs as Ipilimumab is an atypical but renowned eventuality. Awareness of this scenario and its radiological features helps the Radiologist to avoid misdiagnosis of disease progression. In this case report, we present a patient operated for advanced cutaneous melanoma of the left forearm who developed hilar adenopathies with lung and splenic nodules during therapy with Ipilimumab in adjuvant setting. These findings were at first referred to as disease recurrences. Based on discrepancies between imaging, clinic and blood test findings we decided to put the patient on strict follow-up which showed a spontaneous complete regression on the visceral lesions few months after Ipilimumab withheld.

## Case presentation

**Figure 1.**
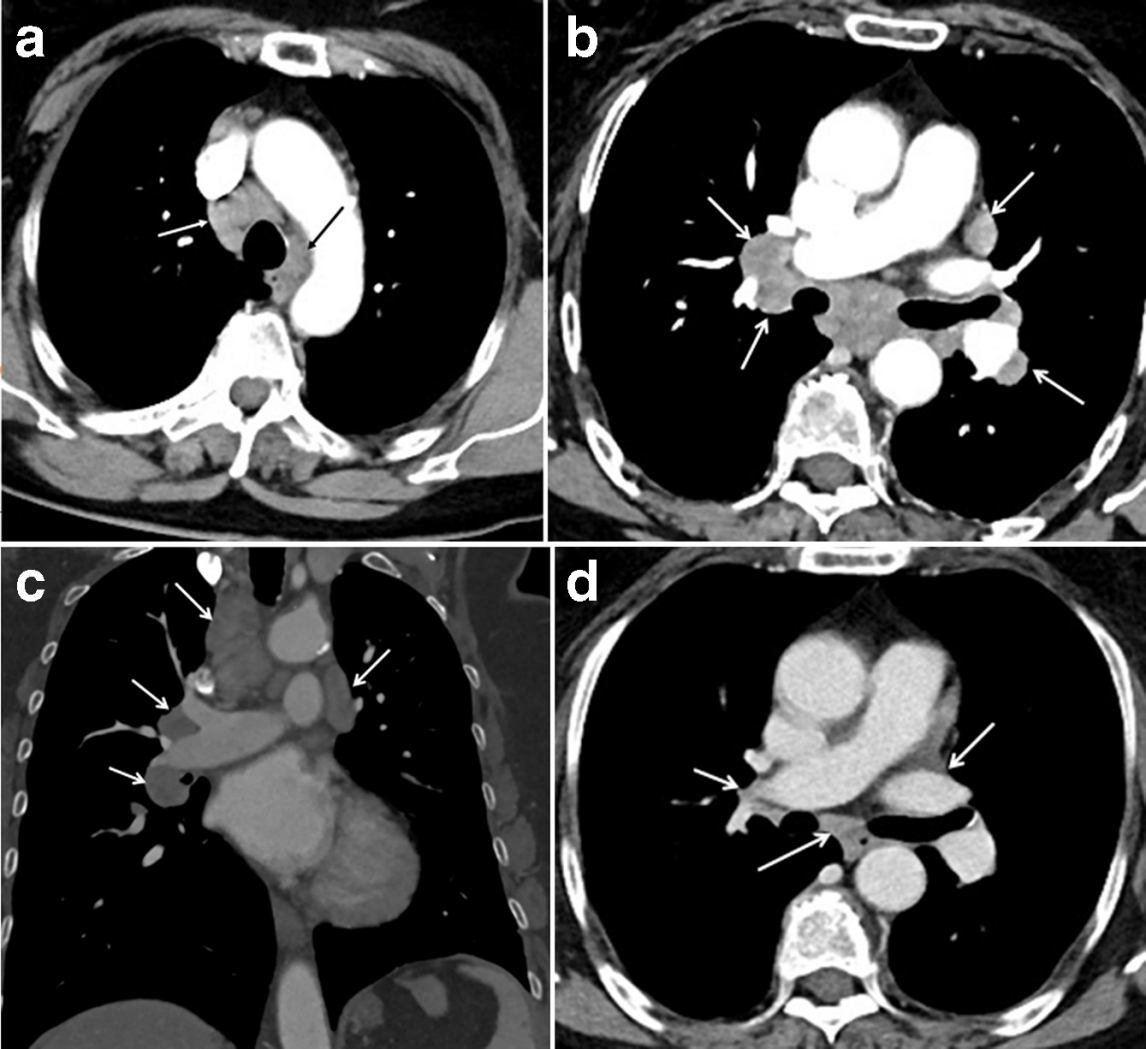
Axial (a–b) and coronal (c) thoracic contrast-enhanced CT scan shows pathological enlarged in the paratracheal (arrows) and hilar region (arrows); these findings define the 1–2–3 sign or Garland triad on chest radiographs. Lymph nodes appeared 3 months after the beginning of Ipilimumab therapy and lasted for 18 months before disappearing spontaneously after 9 months from Ipilimumab discontinuation (d).

**Figure 2.**
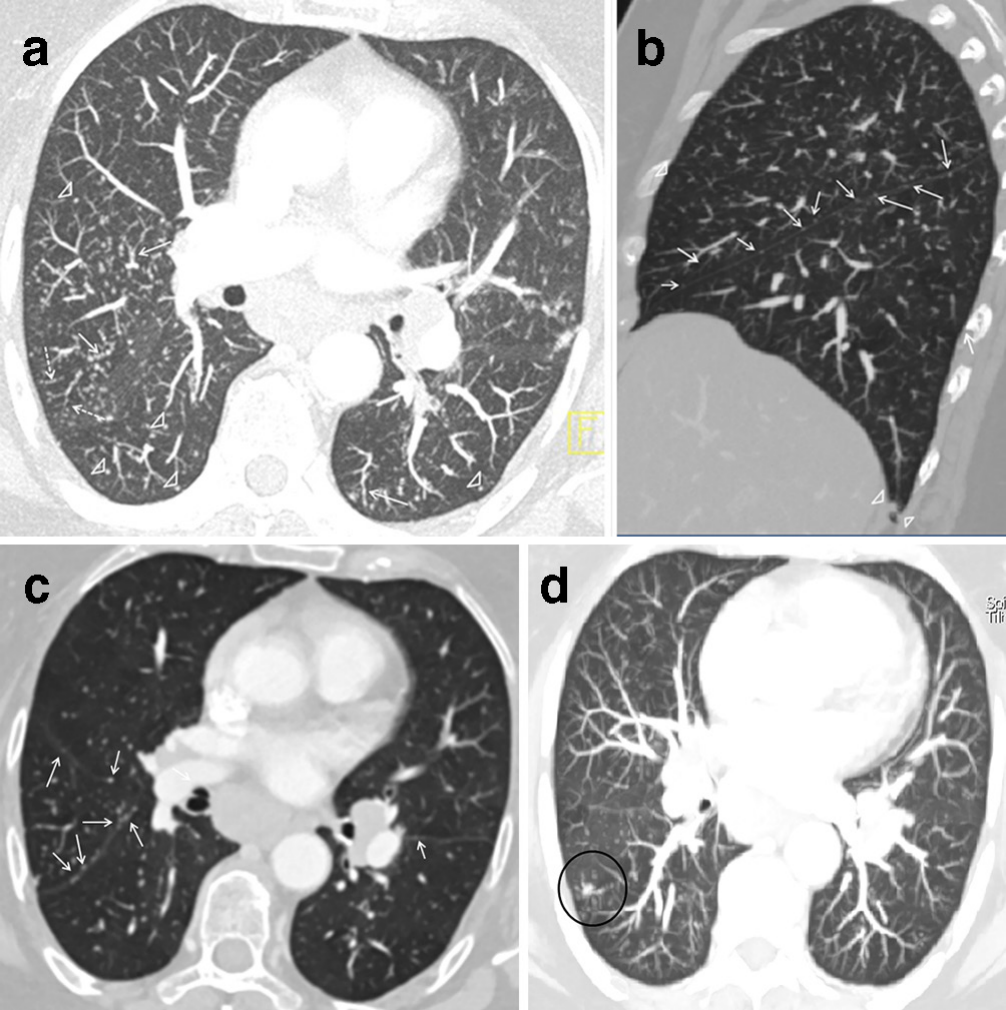
Contrast-enhanced CT scan of the thorax shows unnumbered small lung micronodules with predominance of middle/upper lungs with perilymphatic and miliary pattern of distribution. (a) Perilymphatic nodular thickening along bronchovascular bundle (arrow) and centrolobular nodes (arrow-heads) define central interstitium involvement; dotted arrows highlight interlobular septa nodules suggestive of peripheral interstitium involvement (b–c) Nodulations along lobar fissures (arrows) and in subpleural regions (arrow-head) also define peripheral interstitium involvement. (d) The circle highlights a typical appearance of galaxy sign *i.e*. a large central nodule surrounded by smaller nodules.

**Figure 3.**
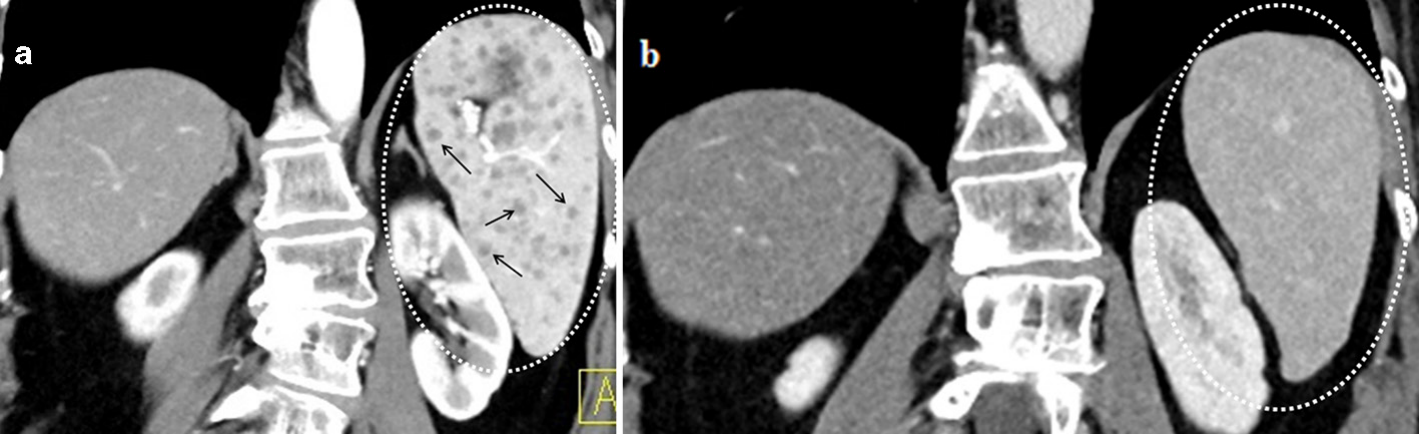
(a) Abdominal contrast-enhanced CT scan showed unnumbered subcentimetric focal spleen lesions (the most significant findings were reported with arrows) and splenomegaly (dotted circle) of 13 cm arised after 11 months of therapy with Ipilimumab. Spleen hypodensities lasted for 10 months circa and then spontaneously disappeared. (b) In April 2017, no more splenic lesions were detectable (dotted circle).

**Figure 4.**
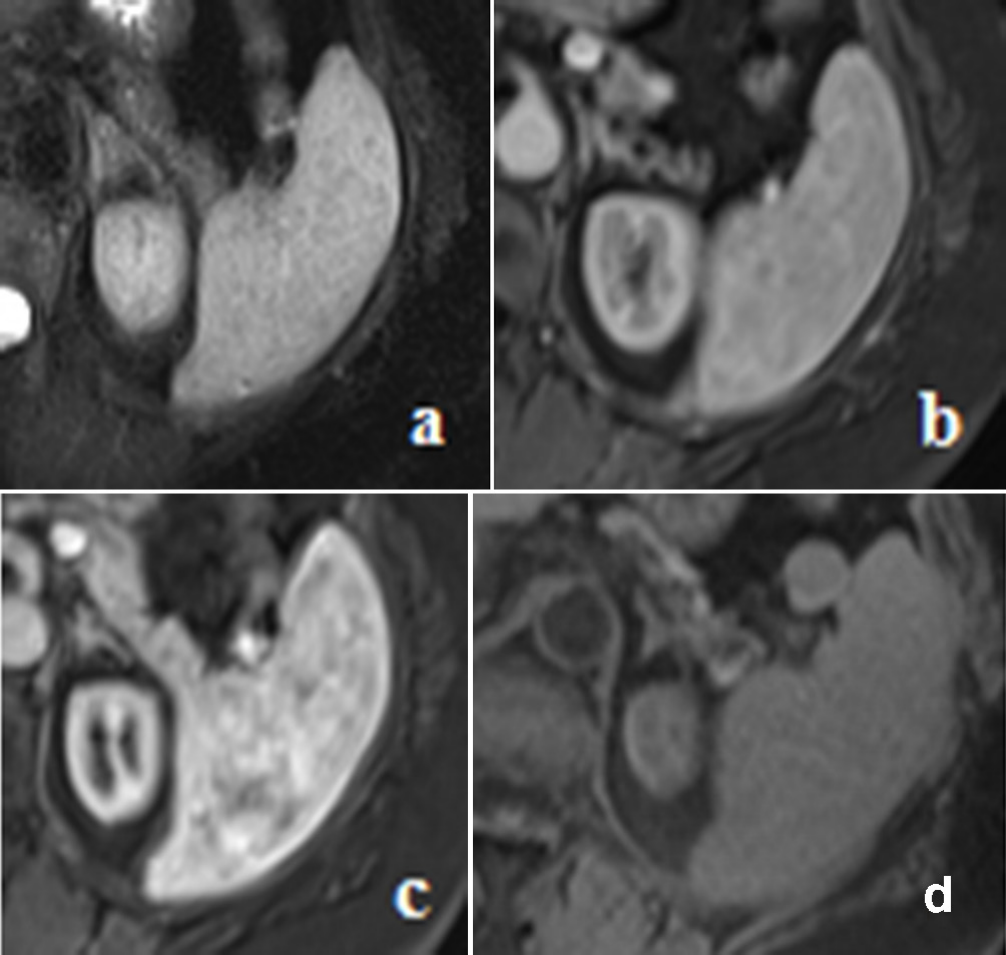
(from left to right): Abdominal MRI with paramagnetic contrast medium: no splenic nodules were clear on *T*_2_ sequence (a); feeble spleen nodules are visible on *T*_1_ images (b); splenic nodules show no contrast enhancement both in the arterial (c) and in the venous (d) phase after contrast medium intravenous injection.

**Figure 5.**
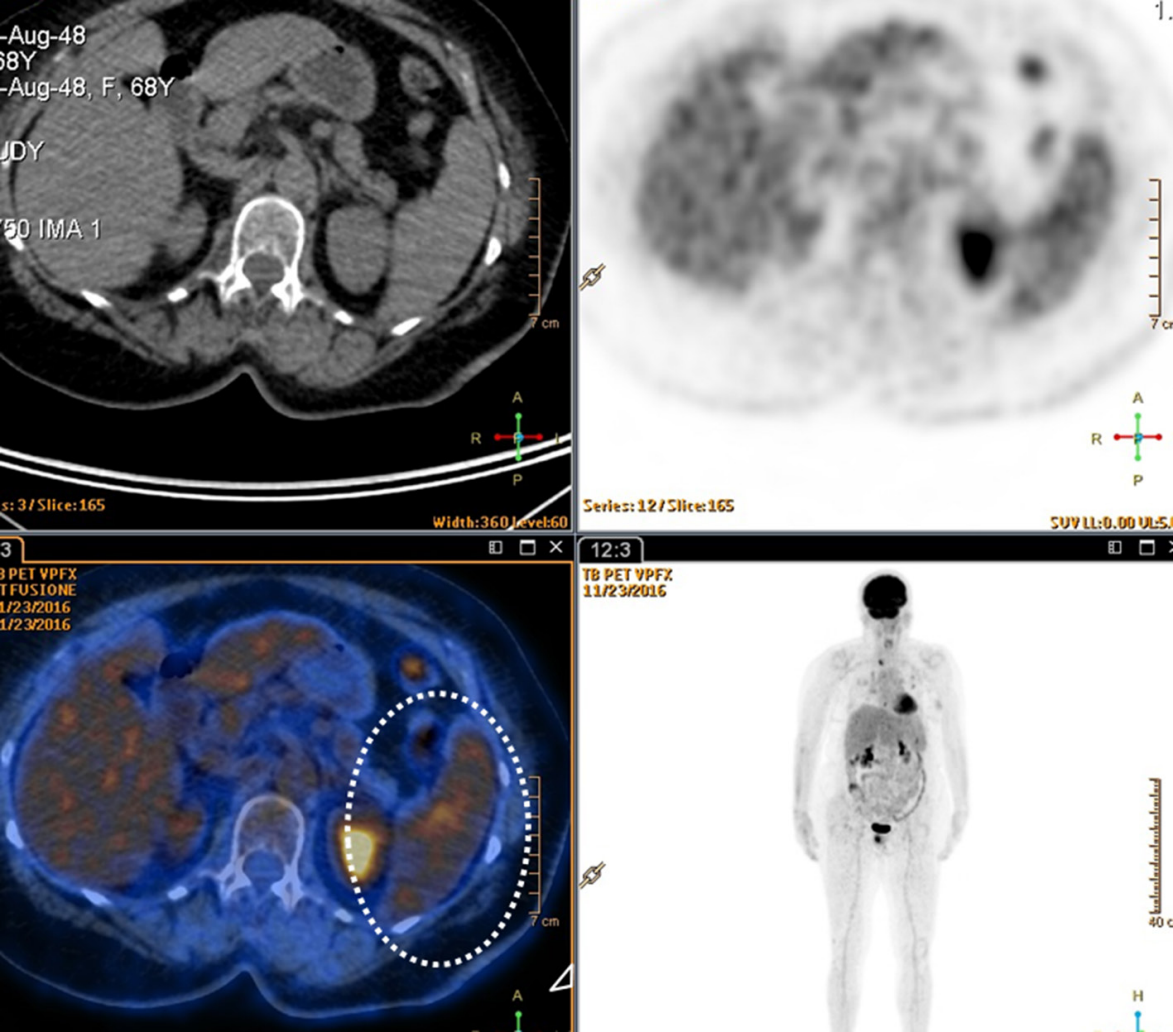
Splenic nodules show no increased uptake of radiopharmaceutical at positron emission tomography with ^18^F-labeled flu-2-deoxyglucose; in particular it is evident in the fusion image (dotted circle).

**Figure 6.**
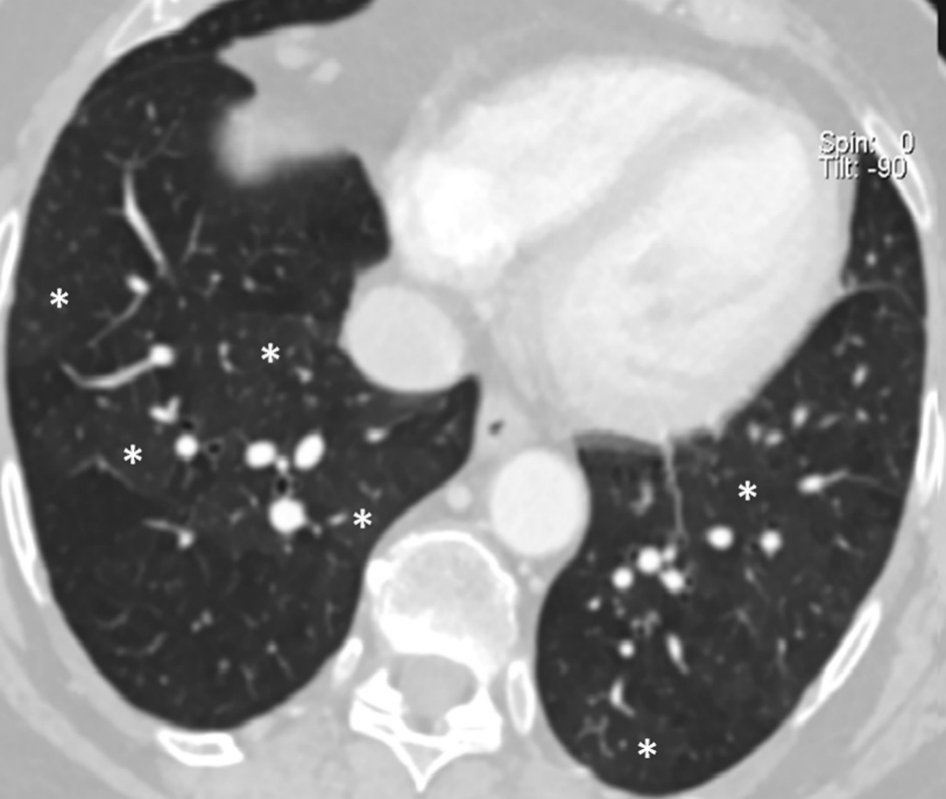
Thoracic CT-scan in inspiration: 1 year after the onset lung micronodules spontaneously receded without any clear chronic parenchymal sequelae (*e.g.* fibrosis); a feeble mosaic attenuation pattern areas (stars) persists, probably attributable a granulomatous bronchiolitis a well-known manifestation of pulmonary sarcoidosis

In February 2014, a 66-year-old female patient underwent locoregional surgery to remove a nodular melanoma (Breslow thickness 3.5 mm, Clark's Level IV, non-ulcerated, 1–6 mitosis/mm) from the left forearm followed by an ipsilateral axillary lymph-node dissection (2 on 20 lymph-nodes were positive for malignant cell). A subsequent total body CT scan (CTs) showed no distant metastasis leading to a final TNM staging of pT3N1aM0. 1 year later ca., in June 2015, disease relapsed with two massive left subclavian nodal metastasis treated with local excision. After surgery, the patient agreed to join an institutional clinical trial with intravenous Ipilimumab administration (10 mg/kg) as adjuvant therapy for 1 year. The first dose of Ipilimumab was administered on 03/08/2015 and the last dose on 07/07/2016 for a total of 51 administrations; the patient withheld Ipilimumab administration during this period just two times for personal reasons. After 2 month of immunotherapy, in October 2015, the patient developed multiple hilar lung adenopathies (Figure 1a–b–c) and, from April 2016, the patient suffered from numerous lung micronodules with perilymphatic and miliary distribution with predominance in the mid/upper lobes bilaterally. Perilymphatic distribution is characterized by the involvement of central and peripheral interstitium. Centrolobular nodules and small micronodules along the bronchovascular bundles are typical features of central interstitium involvement while micronodules in the subpleuric regions or along interlobar fissures and interlobular septa are attributable to peripheral interstitium implication (Figure 2a–b–c). Moreover, mostly in the peripheral regions, several micronodules showed the tendency to converge in a single pulmonary nodule; these types of nodules are characterized by a very dense center and by more rare micronodules in the periphery resulting in an irregular nodular border leading to the typical galaxy sign found in sarcoidosis (Figure 2d). As the patient referred intermittent mild hacking cough, mild dyspnea and mild malaise with low-grade fever, the findings were interpreted as lung marks of a inflammatory episode and the patient continued the usual instrumental follow-up. Afterwards, on July 2016, a scheduled restaging CT scan showed important splenomegaly, with bipolar diameter 13.5 cm, and the onset of outnumbered splenic subcentimetric hypodensities evocative for metastatic lesions (Figure 3a–b). A contrast-enhanced abdominal MR was then performed to characterize the lesions: splenic nodules were hypointense on *T*_2_ weighing and scarcely clear on *T*_1_ weighing without any contrast enhancement on the dynamic sequences (Figure 4a–b–c–d). These MR findings were equivocal and attributable both to an inflammatory or hematologic process or to metastatic lesions. Blood exams showed no significant modifications, especially LDH, so the patient performed a positron emission tomography (PET) with ^18^F-labeled fluoro-2-deoxyglucose (FDG), that showed no pathological uptakes all over the body (Figure 5). Based on radiological, clinical and blood test data radiologists then suspected a sarcoidosis-like scenario as an immune-related adverse event (irAE) triggered by Ipilimumab. Therefore, at the multidisciplinary meeting of the melanoma board, we decided to avoid any invasive procedure or splenectomy and to keep the patient on strict instrumental follow-up. 1 month later, the patient underwent another contrast-enhanced abdominal MR that showed disease stability as long as subsequents instrumental follow-up with contrast enhanced CTs. On November 2016, bilateral hilar adenopaties spontaneously started to reduce and disappeared on April 2017 (Figure 1d), as long as the bilateral lung nodules (Figure 6). To be noticed that bilateral faint mosaic attenuation pattern areas persisted in the lungs for a further 4 months before disappearing completely. In the first instance this manifestation was attributed to a small airway disease, in all probability a granulomatous bronchiolitis, a well-known manifestation of pulmonary sarcoidosis. Finally, on restaging CT scan performed in January 2017, no more splenic lesions were clear. It must be specified that the patient did not suffer from any autoimmune disease, such as sarcoidosis, before starting Ipilimumab therapy.

## Treatment

No specific treatment was necessary. The patient remained slightly symptomatic (slight cough and fever) during the sarcoidosis flare and the granulomatous visceral lesions receded spontaneously 6 months after the last Ipilimumab administration. Also the patient continued Ipilimumab until prescheduled drug discontinuation following trial’s directives.

## Outcome

As of June 2019, the patient is alive without any radiological, clinical or laboratory sign of sarcoidosis or disease relapse. She continues her usual clinical and instrumental oncologic follow-up.

## Discussion

Melanoma is a relatively rare skin cancer involving melanocytes which affect mostly youngsters and young adults and is the second and third more frequent tumor in males and females respectively at 0–49 range of age.^1^ It is an aggressive disease scarcely responsive to chemotherapy and radiation therapy. Melanoma is a highly immunogenic tumor and it is characterized by a complex neoantigen scenery that plays a crucial role in modulating anti tumor activity of T-cells and in response to Check-Point inhibitors (CPi) therapy, such as Ipilimumab. Solid scientific data^2,3^ show a significant improvement of overall survival (OS) with durable responses in patient affected by metastatic melanoma treated with Ipilimumab. Ipilimumab is a fully humanized antibody direct against T-lymphocyte-associated protein 4 (CTLA-4). Tumor cells are able to neutralize T-lymphocytes surveillance favoring a binding between CTLA-4 and CD86 on activated T cells. This bond induce an anergic state of T-lymphocytes protecting tumor cells from immune-mediate destruction.^4^ Ipilimumab attempts to restore patient’s immune function blocking this bond and strengthening T-lymphocyte natural activity against malignant cells reactivating a Th-1 helper immune response. It also increases blood levels of proinflammatory compounds mostly IL-2, IL-6, IFN-ƴ and TNF-α.^5^ This cytokines-related proinflammatory action is supposed to trigger immune-related Adverse Events (irAE) like colitis, diarrhea, hypophysitis, hepatitis, uveitis and dermatological problems such as dermal hypersensitivity reactions, lichenoid eruptions, immunebullous reactions and vitiligo.^6,7^ Moreover, the mechanisms of action it is self-sufficient since the increased lysis of melanoma cells exposes additional neoantigens to antigen-presenting cells. It may foster a Th-1 response with the consequent establishment of a proinflammatory cytokine environment favorable to develop irAE during CPI therapy.

Among irAE, granulomatous/sarcoidosis-like reactions in particular are decidedly rare with a slight female predominance (M:F ratio 0,85:1) but are clinically and radiologically significant, impacting both on the disease recurrence misdiagnosis and on the treatment of the patients. IrAEs can be clinically very relevant and have a strong impact on the patient's quality of life as they may need hospitalization and/or new drugs to control their progression. Ultimately, they may compel the clinician to decide immune-therapy discontinuation in order to resolve the adverse events. Others CPi like Nivolumab, Pembrolizumab and others anti programmed cell death protein one ligand drugs share similar biological and molecular pathways leading to similar irAE.^8^

Sarcoidosis is an aberrant multisystemic, inflammatory, non-caseating granulomatous disease of unknown origins initiated by T-helper 1 cells secreting interleukin-2 and interferon-γ, leading to the activation of additional T cells and macrophages. It is triggered by several environmental causes like infections, chemical agents or drugs in genetically predisposed patients and remains a diagnosis of exclusion.^9^ Granulomata can occur all over the body, most commonly in skin, lungs and lymph-nodes with other important involvements in heart, eyes and nervous system and a presumptive diagnosis of sarcoidosis may be made on the basis of clinical and radiographic features.^10^ A sarcoid-like reactions (SLRs) is referred to localized clinical and radiological features without fulfilling the sarcoidosis criteria.

A SLRs shown in our paper is a very uncommon and atypical irAE during CPi therapy^8^ with scarce literature as to its imaging appearance and appropriate management. In particular, splenic involvement is anecdotal in literature. In 2009, Eckert et al described for the first time a sarcoidosis-like spleen involvement during Ipilimumab therapy in melanoma.^11^ Since that time, few others studies showed the correlation between Ipilimumab treatment and development of lung and splenic sarcoidosis.^12,13^ Presence of pulmonary sarcoidosis nodules has been recently proved also as an autopsic finding in a patient treated with CPi.^14^ In 2018, Tetzlaff et al reviewed literature about SLRs development during CPi therapy. Focusing on melanoma therapy with Ipilimumab, they found 20 cases of patient with sarcoidosis-like scenario and, while skin, pulmonary and nodal sarcoidosis nodules were more common findings, splenic involvement is a rare and infrequent circumstance in this setting of patients^15^; moreover our patient didn’t suffer from any cutaneous hypersensitivities reaction (*e.g.* dermal panniculitis, skin nodules) unlike many patients reported by Tetzlaff et al. In literature, the median duration of CPi therapy in patients who developed SLRs was 6 months and the treatment included mostly CPi therapy discontinuation (38%) with or without systemic steroids administration (44%). However. in most of the cases, the SLR presented mostly a benign, uncomplicated disease and a partial or complete resolution of these types of irAE is obtained in 96% of patients irrespective of how toxicity was managed by clinicians. Furthermore, data in the literature show that the development of a SLR could be correlated with a better tumor response to treatment with CPi in a subset of patients.^11–16^

Nowadays, Ipilimumab and other CPi drugs are widely used as standard therapy to cure different oncological diseases and will become increasingly important in the future. Therefore it is crucial, not only for clinicians, but also for radiologists, to be aware that a sarcoidosis-like visceral reaction could affect patients treated with CPi and these lesions, especially in the spleen, can be easily mistaken for metastases. Moreover, an appropriate framework and recognition of these findings on radiologic reports have a significant impact on patient’s treatment decision.

As in the case report we presented, could it be very hard to distinguish between metastatic lesions and granulomas with conventional instrumental exams like contrast-enhanced CT scan, MRI and FDG-PET, even more so when an aggressive disease like advanced melanoma is implicated. Histology is the gold-standard to discriminate the nature of a nodule, nevertheless we decided to perform a strict follow-up with a watchful waiting sparing the patient from any useless invasive procedures like biopsy or splenectomy.

In the end this case report highlights once again the major importance for radiologists to know the clinical and anamnestic history of the patient while reporting. It would help to avoid misdiagnoses that could lead to unnecessary invasive exams and therapy shifting.

## Learning points

- Patients undergoing anti-CTLA4 drugs like Ipilimumab may develop a sarcoidotic-like radiological scenario with mediastinal lymphadenopathy, lung and splenic nodular involvement.
- This scenario can develop several months after the first intake of anti-CTLA4 drugs and can last for a long time before disappearing spontaneously, without any sequelae.
- Sarcoidotic-like reaction can be also a marker of good proinflammatory response against tumor cells.
- Being aware of this rare irAE can help radiologists to prevent misdiagnosis of disease progression avoiding unnecessary invasive medical examinations or anticancer therapy switch.
- Strict follow-up is crucial for a safe and correct management of patients in this setting.
